# Frequency of Topical Immunomodulatory and Immunosuppressive Therapies for Ocular Chronic Graft-versus-Host Disease

**DOI:** 10.3390/jcm13164728

**Published:** 2024-08-12

**Authors:** David Sinan Koca, Tina Dietrich-Ntoukas

**Affiliations:** Department of Ophthalmology, Charité—University Medicine Berlin, Corporate Member of Freie Universität Berlin, Humboldt Universität zu Berlin, and Berlin Institute of Health, Augustenburger Platz 1, 13353 Berlin, Germany; david-sinan.koca@charite.de

**Keywords:** ocular chronic graft-versus-host disease, topical immunomodulatory/immunosuppressive therapy, steroid eye drops, cyclosporine A eye drops, topical therapy

## Abstract

**Introduction:** The purpose of the study was to evaluate the frequency of topical immunomodulatory and immunosuppressive therapies in patients with ocular chronic graft-versus-host disease (cGVHD) in consideration of inflammatory activity and systemic immunosuppressive therapies in a tertiary care university hospital setting. **Methods:** We included 95 adult patients (48 male, 47 female) with ocular chronic graft-versus-host disease (cGVHD) after alloHSCT (median age 49.5 years). Clinical ophthalmological findings and the grade of ocular cGVHD according to the NIH eye score and the German–Austrian–Swiss Consensus (GAS) Grading were analyzed. Systemic GVHD manifestations as well as the prevalence of topical and systemic (immunomodulatory) therapies were assessed. **Results:** A total of 74 of 95 patients (77.8%) had manifestations of systemic chronic graft-versus-host disease other than ocular GVHD. 68.42% (65/95) of patients were under systemic immunosuppressive therapy with at least one immunosuppressive medication. All patients (95/95) received lid-margin hygiene and phosphate- and preservative-free lubricating eye drops. Twenty-five percent of the cohort (24/95) were treated with autologous serum eye drops (ASEDs). In total, 80% (76/95) of patients required topical steroid therapy to treat acute exacerbation of inflammation at least once; continuous topical steroid therapy was only necessary for a minor part (12%) with refractory chronic inflammation. A total of 92.63% (88/95) were primarily treated with ciclosporin A 0.1% as Ikervis^®^, of whom at least one third did not continue the therapy because of intolerable side effects during follow-up and received alternative topical formulations. **Conclusions:** Our data show that patients with ocular cGVHD mostly need topical therapy including anti-inflammatory agents despite systemic immunosuppressive therapy. In our cohort, 80% of patients received topical steroids, and more than 90% received topical ciclosporin A eye drops, which were tolerated by only two thirds of patients due to side effects.

## 1. Introduction

Graft-versus-host disease (GVHD) is a common cause of morbidity and mortality after allogeneic hematopoietic stem cell transplantation (alloHSCT) [1,2]. Ocular involvement is one of the most frequent organ manifestations after alloHSCT and mostly accompanies other chronic GVHD (cGVHD) manifestations: 60–80% of patients with GVHD involvement of other organs suffer from ocular chronic GVHD. However, ocular cGVHD can also be the only manifestation of GVHD in some patients.

Ocular cGVHD can affect different tissues of the eye [3], but is mainly an ocular surface disease: the involvement of the lacrimal glands (inflammation, obstruction, atrophy, decreased tear production), the conjunctiva (apoptosis of goblet cells, inflammation, fibrosis), the lids including the meibomian glands (obstruction, hyperkeratosis, blepharitis), and the cornea (punctate keratopathy, filamentary keratitis, corneal erosions and ulcers, superior limbic keratitis) leads to a severe disorder of the ocular surface [4,5]. The ocular surface disease in cGVHD is often irreversible and can be a cause of vision loss, which is mainly related to corneal complications like persistent or recurrent erosions, corneal calcification, corneal scarring and thinning, ulcers, and corneal melting [6,7]. Quality of life can be substantially affected by ocular GVHD [8].

GVHD is an immune-mediated disease, which primarily involves T cells, but also macrophages and B cells [9]. These inflammatory processes, apoptosis, and fibrosis are currently thought to mainly cause dry eye and ocular surface disease in cGVHD [10,11]. The binding of the host’s specific antigens to the T-cell receptors of the donor plays a crucial role in the pathogenesis of GVHD [12]. Calcineurin is important in this pathway of T-cell activation; its inhibition by calcineurin inhibitors such as cyclosporine A and tacrolimus is therefore an effective therapeutic strategy in GVHD [12]. T-cell-related processes, different cytokines, and integrins (as cell adhesion and signaling molecules) play a role in ocular chronic GVHD. The migration of T cells depends on the binding of LFA-1 to ICAM-1, which can be blocked by lifitegrast [13]. Different Th-1-associated chemokines (chemokine C-X-C motif ligand CXCL 9, CXCL10, and CXCL11) and their receptor (chemokine C-X-C motif receptor 3, CXCR3) are upregulated in the conjunctiva of patients with chronic ocular GVHD compared to healthy controls [14]. Tear levels of epidermal growth factor and IP-10/CXCL10 are significantly decreased, while tear levels of interleukin-1Ra, IL-8/CXCL8, and IL-10 are significantly increased in patients with ocular chronic GVHD compared to healthy controls under controlled environmental conditions [15]. Activation of macrophages leads to the release of TGFß, which induces fibrosis of the lacrimal gland and the lacrimal duct [10,11]. Macrophages also secrete IL-1, a proinflammatory cytokine, which can be blocked by IL-1 receptor antagonists, but its therapeutic effect has not been shown in ocular GVHD so far [12]. As it is known for other dry eye diseases, the dryness itself maintains chronic inflammatory processes on the ocular surface [16].

Systemic immunosuppressive therapies can lead to an overall improvement of ocular cGVHD, but are less effective in specific organs such as the mouth, eyes, and genital tract [12,17,18]. Topical treatment is essential in ocular cGVHD for the following reasons: 1. Lubrication of the ocular surface requires topical lubricants because systemic medication is mostly not sufficient [17]. 2. Topical drugs provide higher ocular drug concentrations compared to systemic administration. 3. Topical immunosuppressive drugs may be continued as a treatment for ocular cGVHD and may therefore allow tapering and cessation of systemic immunosuppression [5].

Topical steroids provide high ocular drug concentrations and are able to promote lymphocyte apoptosis and suppress cell-mediated inflammation [19]. Topical steroids (prednisolone acetate 4x per day) have been shown to reduce inflammatory activity in cicatricial conjunctivitis in ocular GVHD and improve related symptoms [20]. They are also indicated in other inflammatory chronic GVHD-related conditions such as superior limbic keratitis (SLK) and episcleritis.

Calcineurin inhibitors such as cyclosporine A (CsA) are immunomodulatory agents that inhibit T-cell activation and action [21]. Therefore, they are an important therapeutic tool in GVHD treatment as described above.

In the United States, cyclosporine 0.05% eye drops were approved by the FDA as Restasis^®^ (Allergan, Irvine, CA, USA) in 2003 and as Cequa^®^ (CSA 0.09%, Sun Pharma, Cranbury, NJ, USA) in 2018 [21,22]. In Europe, cyclosporine A eye drops are only available (a) as a pharmacy-based oil formula (0.05–2%) or (b) as Ikervis^®^ (Santen, Osaka, Japan), a 0.1% (1 mg/mL), sterile, unpreserved, oil-in-water emulsion of CsA, which also contains a cationic agent, cetalkonium chloride (CKC), which increases the resident time of the drops on the ocular surface. Ikervis^®^ is EMA (European Medicines Agency)-approved since 2015 for refractory dry eye disease with corneal involvement [23] and available on the market in Germany. Verkazia^®^ (Santen Oy, Tampere, Finland) is approved by EMA for keratoconjunctivitis vernalis for children > 4 years and on the market in Switzerland - not in Germany—since 2020 [24]. In general, the safety profile of pharmacy-based formulations is lower than that of industrially produced drugs, and the reproducibility and availability are reduced. Access to this topical calcineurin inhibitor is therefore limited in Germany.

In clinical practice and according to published recommendations, topical CSA is included in therapeutic concepts for chronic ocular GVHD [5,25]. Topical cyclosporine eye drops have been shown to be effective in the treatment of dry eye diseases and ocular cGVHD [19,22,26,27]. In randomized clinical trials, it has been shown that CSA eye drops are able to improve the Schirmer’s test and breakup time, increase the number of conjunctival goblet cells, and reduce punctate keratopathy in patients with dry eye disease [28]. However, a recent meta-analysis revealed inconsistent evidence of its effects, mainly because of a lack of long-term studies [29].

The main therapeutic aim of cyclosporine eye drops is a reduction in inflammation and dryness—breaking the vicious circle of dryness and inflammation—resulting in a relief of symptoms, amelioration of ocular findings, and a reduced need for lubricants. Additionally, cyclosporine eye drops are effective in the treatment of blepharitis [30,31], which is also frequently present in patients with chronic GVHD [32]. To date, a recommended dosage of cyclosporine eye drops in GVHD is 0.05% twice daily as long-term treatment [5,25]. A more frequent application (3 to 4 times per day) has been described in refractory patients with ocular GVHD [33] as well as the usage of a higher concentration (1%) [27]. The main problem of topical CSA therapy is that a significant proportion of patients with ocular chronic GVHD cannot tolerate the side effects of burning and stinging, blurred vision, swelling, and/or redness of lids and conjunctiva and are therefore not able to continue the therapy for a longer period [34].

The purpose of the present study was to evaluate the frequency of topical immunosuppressive and immunomodulatory drugs in patients with ocular cGVHD with regard to the inflammatory activity of the disease and systemic immunosuppressive therapy in a specialized university care setting. The secondary endpoints were to evaluate (a) the effect of the therapy on the grade of ocular chronic GVHD and (b) the frequency of intolerance of topical calcineurin inhibitors as CSA in a real-world setting.

## 2. Patients and Methods

We executed a retrospective analysis of patients who presented during the years 2016 to 2020 in a tertiary care setting at the outpatient clinic at the Department of Ophthalmology at Campus Virchow-Klinikum of Charité—University Medicine Berlin, Berlin, Germany. The study was performed according to the Declaration of Helsinki and approved by the local ethics committee (EA2/071/24). Patients were referred either by the treating hemato-oncologists or ophthalmologists, either from the same region or from other regions of the country. All patients were seen and graded by a senior ophthalmologist with more than 20 years’ experience in ocular cGVHD (TDN).

Ophthalmological examination included best corrected visual acuity (BCVA), intraocular pressure (Goldmann’s applanation tonometry, Haag-Streit, Bern, Switzerland, or pneumatic tonometer, CT20D, Topcon, Tokyo, Japan), anterior segment slit lamp examination (including subtarsal inspection of the conjunctiva and fluorescein staining of the ocular surface), fundus examination, Schirmer’s test and tear film breakup time (BUT) as described previously [32]. Schirmer’s test was performed by placing sterile strips of filter paper in the lower fornix and leaving it in place for 5 min with eyes closed without anesthesia [18]. Both eyes were tested simultaneously, and the wet portion of the strip was measured in millimeters. The breakup time of the tear film (BUT) was measured after using fluorescein strips: patients were asked to blink several times and then to keep their eyes open. The cornea was then examined using a cobalt blue filter on the slit lamp and dry areas were detected as black spots. Tear film breakup time was recorded in seconds as the time between the last blink and the appearance of a random dry spot.

According to the published diagnostic recommendations, ocular cGVHD was diagnosed using the following criteria: distinctive manifestations of chronic GVHD include new onset of dry, “gritty”, or painful eyes, cicatricial conjunctivitis, keratoconjunctivitis sicca (KCS), and confluent areas of punctate keratopathy in the slit lamp examination; diagnosis requires Schirmer’s test < 5 mm or biopsy [5]. Other not distinctive, but typical features include photophobia, periorbital hyperpigmentation, and blepharitis (erythema and edema of the eyelids and telangiectasia of the lid margin). A positive Schirmer’s score less than 5 mm or a Schirmer’s score ranging from 6 to 10 mm plus the new onset of keratoconjunctivitis sicca with typical signs in the slit lamp exam is necessary to diagnose ocular GVHD in patients who have GVHD involving at least one or more organs [35].

All patients were classified using the grading scale, which was proposed by the German–Austrian–Swiss (GAS) Consensus Conference on Clinical Practice in cGVHD [5] and validated [32]: it includes tissue involvement, inflammatory activity, presence of complications, and functional impairment [Table 1]. Inflammatory activity of ocular GVHD was graded at each visit: inflammation of the conjunctiva was classified according to the proposed GAS Consensus Conference grading as none, mild, moderate, or severe [5], and punctate keratopathy was graded into none (grade 0), mild (grade I/II), moderate (grade III), or severe (grade IV) according to Blecha et al. [32]. Additionally, all patients were graded according to the NIH Consensus recommendation by means of the NIH eye score [18,35] Table 2 at initial presentation and at first and last presentation during topical therapy. The NIH eye score has been validated as a sensitive measurement scale and response assessment for chronic ocular GVHD [36,37].

Other ophthalmological diseases such as cataracts and glaucoma (alloHSCT-related or non-related) were documented. Subjective symptoms of keratoconjunctivitis sicca (KCS, dry eye disease, DED) and the use of eye drops as well as other treatments and systemic medications were assessed.

Therapy was analyzed in a detailed manner: the kind and duration of systemic and topical immunomodulatory and immunosuppressive therapy (including topical steroids, cyclosporin A in different formulations, tacrolimus), use of lubricant eye drops, punctum plugs, and (autologous) serum eye drops (ASEDs) as well as other medications (e.g., anti-glaucomatous eye drops). All patients were informed about possible side effects of the topical therapy, e.g., elevated pressure for topical steroids and possible side effects, especially at the beginning of using topical calcineurin inhibitors, such as stinging, burning, redness, swelling, etc. Whenever indicated by inflammatory activity, therapy was started with topical steroids—as short-term therapy—and calcineurin inhibitors—as long-term therapy—at the same time in order to reduce possible side effects of the latter. Patients were informed about the therapeutic effect being expected not earlier than several weeks after the start of therapy. Drug intolerance was defined as cessation of the therapy by the patient due to intolerable side effects such as stinging burning, redness, swelling, and visual disturbances such as blurred vision.

We collected the epidemiological data concerning age, sex, reason (underlying disease) for alloHSCT, systemic manifestations of graft-versus-host disease, and systemic immunosuppressive therapy. Data were gathered exclusively from the digital documentation system SAP and rendered anonymous for further analysis. Analyses were performed with MS Excel version 2303 and SPSS version 25 with pairwise deletion. Parameters are either presented as mean (SD, min–max) or median (SD, min–max).

## 3. Results

We included 95 adult patients (48 men, 47 women) with chronic ocular graft-versus-host disease (GVHD) after alloHSCT. The median age was 49.5 years (*SD* = 12.11 years, range 27–82 years). The main indication for alloHSCT was leukemia (71%), with AML (acute myeloic leukemia) (35%) and ALL (acute lymphatic/lymphoblastic leukemia) (13%) being the most common. Other reasons for alloHSCT were lymphoma (19%) and myelodysplastic syndrome (14%); diagnoses are listed in Table 3. Seventy-four patients (77.8%) had manifestations of chronic graft-versus-host disease other than ocular GVHD. This comprised mostly skin (58.95%), oral and nasal mucosa (31.58%), gastrointestinal tract (22.11%), liver (20.00%), lung (12.63%), and in one case joint synovia (3.16%) (Table 4).

### 3.1. Systemic Immunosuppressive and Immunomodulatory Drugs

In total, 68.42% (65/95) of patients were under systemic immunosuppressive therapy. 34,74% (33/95) under monotherapy: 17.89% (17/95) were treated with systemic CSA and 9.47% (9/95) with oral prednisolone; other regimes included sirolimus (3/95), tacrolimus (1/95), mycofenolat mofetil (2/95), ruxolitinib (2/95) and lenalidomide (1/95).

A total of 30.5% (27/95) of patients were under two systemic immunosuppressive drugs, with the combination of ciclosporine A and prednisolone being most common (19/95), followed by ciclosporin A and mycofenolate mofetil. 5.26% (5/95) received immunosuppressive triple therapy, e.g., ciclosporin, prednisolone, and ibrutinib; for details, see Table 5.

### 3.2. Ophthalmological Examination Parameters

BCVA of the patients at initial presentation ranged between +3.0 logMAR (light perception) and −0.1 logMar (=1.25). BCVA at last presentation ranged between amaurosis and −0.1 logMAR (=1.25). Intraocular pressure was between 9 and 25 mmHg at first presentation. At initial presentation, 89.47% (85/95) of patients showed lacrimal gland dysfunction with pathological Schirmer’s test (<10 mm) and/or tear film instability with reduced BUT (<10 s). Additionally, 75.79% (72/95) of patients had eyelid involvement (erythema, meibomian gland dysfunction, blepharitis), and 94.74% (90/95) of patients suffered from conjunctival involvement including redness, chemosis, conjunctival erosion, and lid-parallel conjunctival folds. In total, 84.21% (80/95) of patients showed corneal pathology (including superficial punctate keratitis, erosion, ulceration). Thirty-seven percent (36.84%; 35/95) of patients had other ocular problems including cataracts, ectropium, tear duct stenosis, or uveitis. Twenty percent of patients suffered from complications that led to irreversible functional impairment including corneal perforation, corneal scarring, and advanced symblephara.

During the time of documentation, 25.3% of patients developed relevant cataracts, mainly (52%) subcapsular cataracts. Twelve percent of patients suffered from corneal pathologies such as scarring or ulceration, six percent of patients had herpes simplex keratitis and two percent needed emergency keratoplasty surgery. Five percent of patients had stenosis of the lacrimal duct, and four percent of patients reportedly suffered from glaucoma (Table 6). During the documented period, several surgical interventions were necessary, e.g., for cataract or tear duct stenosis.

### 3.3. Grading: NIH Eye Score and GAS Consensus Score

In total, 81% (61/75) of patients who underwent at least one follow-up examination displayed a moderate (52%) to severe (29%) ocular cGvHD according to the NIH eye score. The change in NIH eye score from the beginning to the end of follow-up is shown in Table 7. Grading of inflammatory activity according to the German–Austrian–Swiss (GAS) grading proposal revealed moderate to severe inflammatory activity in 48% (36/75) of patients.

Before receiving treatment with Ikervis^®^, 30% of patients (22/75) suffered from severe ocular cGvHD and 53% of patients (39/75) suffered from moderate ocular cGvHD according to the NIH eye score. When therapy with an alternative CSA formulation (AF) was initiated, only 19% (3/16) of patients showed a severe and 31% (5/16) moderate ocular cGvHD. Concerning the therapy with ASEDs, 27% (6/22) displayed severe and 54% (12/22) moderate disease.

### 3.4. Frequency and Tolerance of Topical Therapy

All patients (95/95) received lid-margin hygiene and lubricating eye drops, mainly phosphate- and preservative-free. In total, 25% (24/95) were treated at least once (for 3 months) with autologous serum eye drops (ASEDs), and 20% (19/95) had at least one follow-up examination under ASED therapy. In total, 83% (20/24) received ASEDs in addition to topical therapy with CSA (either Ikervis ^®^ or AF), 17% (4/24) received ASEDs without CSA (despite indication) due to intolerance to CSA formulations, and 26% (25/95) received punctum plugs during the documented period (Table 8).

The mean duration of treatment in our department was 31.3 months (SD = 30.6 months, range 4 weeks–183 months; Table 9). In total, 92.63% (88/95) were treated with cyclosporine A 0.1% as Ikervis^®^, and 78.95% (75/95) underwent at least one follow-up examination under therapy with Ikervis^®^ (Table 8). 33,33 % (25/75) did not continue the therapy with Ikervis^®^ during the further follow up because of intolerable side effects. Documented symptoms and signs of intolerance were burning, redness, swelling of the lids, chemosis of the conjunctiva, visual disturbances, and inflammatory reaction of the oral mucosa. Of these patients, 96% (24/25) received an alternative topical immunomodulatory therapy.

In total, 25.26% (24/95) received an alternative pharmacy-based formulation (AF) at least once, and 2.11% received (2/95) two alternative preparations due to intolerance to the first given drug. A total of 66.67% (16/24) of patients receiving an alternative pharmacy-based formulation were treated with CSA in castor oil, 41.67% (10/24) with CSA in liposomal solution and one patient with tacrolimus eye drops 0.02% as an alternative calcineurin inhibitor. Four percent (4/95) were primarily treated with a pharmacy-based formulation of CSA (Table 8).

**Figure 1:**
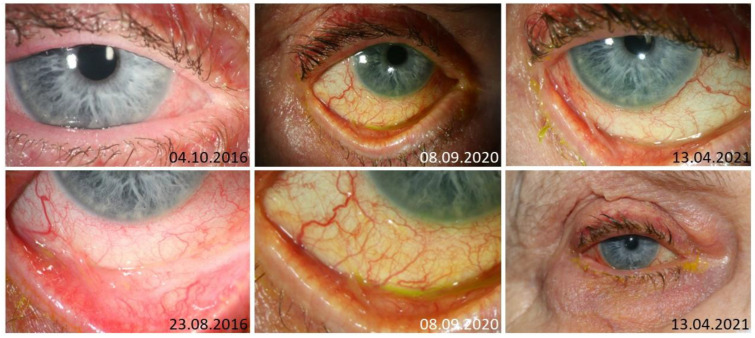
Slit lamp images of an 81-year-old female patient with ocular chronic GVHD with involvement of lids, conjunctiva (including symblephara), and cornea (punctate keratitis) and ongoing inflammatory activity despite systemic and topical immunomodulatory treatments. Because of the side effects of commercially available CSA eye drops, alternative cyclosporine A formulations in different concentrations and tacrolimus eye drops were prescribed, but showed no effect on the inflammatory activity; the patient received prolonged topical steroid therapy.

Eighty percent (76/95) of the patients required at least one period of topical steroid therapy to treat acute exacerbation of inflammation. Indications were acute inflammatory episodes (with higher grades of inflammation than documented before) or ongoing inflammatory activity despite immunomodulatory and/or immunosuppressive therapies. Topical steroid therapy was preferentially applied with non-preserved and phosphate-free preparations and was administered for a limited time period in order to reduce side effects. Hence, in 88.14% (104/118) of the treatment periods, steroid therapy was administered for less than six weeks, mainly three weeks with tapering of the dosage. Continuous topical steroid therapy (>6 weeks; longest documented time period 43 months) was only necessary in a minor part (11.86%) and was administered exclusively in cases of severe refractory chronic inflammation (Figure 1). For details of topical steroid therapy, see Table 9 and Table 10.

### 3.5. Clinical Course under Therapy with CSA 0.1% as Cationic Emulsion (Ikervis^®^)

**Figure 2:**
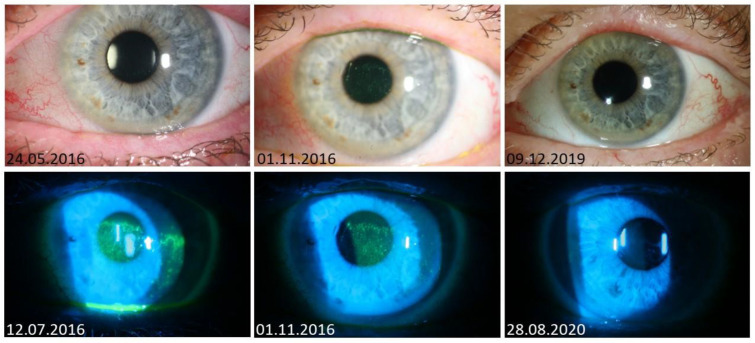
Slit lamp images of a 59-year-old female patient with ocular chronic GVHD with good response to Ikervis^®^. After 6 months of therapy, the inflammatory surface disease decreased with a reduction in conjunctival hyperemia and punctate keratopathy. In March 2019, the patient stopped Ikervis^®^ by herself and continued only systemic immunosuppression with ibrutinib according to the treating hemato-oncologists.

The mean duration of therapy with Ikervis^®^ was *M* = 18.7 months (*SD* = 16.7 months; range 4 weeks–61 months). Seventy-nine percent (75/95) of patients underwent at least one follow-up under therapy with Ikervis^®^. Before induction of a multimodal therapy including Ikervis^®^, most patients showed moderate (53%) to severe (30%) disease according to the NIH eye score. After therapy or at last presentation during the follow-up period, 96% of patients showed a stable disease or improvement (Figure 2), and most patients (64%) showed mild disease. Regarding BCVA, 41.33% (31/75) had an increase in BCVA, 24% (18/75) had a stable BCVA, and 34.67% (26/75) had a decreased BCVA. In terms of inflammatory activity according to the GAS criteria, we saw a reduction in ocular surface inflammation during the follow-up period. For details, see Table 7, Table 11 and Table 12.

### 3.6. Clinical Course under Therapy with Alternative Pharmacy-Based Formulations (AFs)

The mean duration of therapy with AF was *M* = 17.0 months (*SD* = 16.0 months, range 1 month–59.0 months). 20/95 underwent at least one follow-up under AF therapy; for 16 of these patients NIH eye scores were completely documented (Table 11). Two percent (2/95) needed to change AFs due to drug intolerance: one patient received CSA in liposomal solution after not tolerating CSA in castor oil. Another patient formerly treated with CSA in liposomal solution started treatment with CSA in castor oil. When first receiving therapy with AFs, most patients showed mild to moderate disease; after therapy with AFs (or at the last presentation during follow-up), most of the patients had reduced NIH eye scores as shown in Table 10 and reduced inflammatory activity according to the GAS consensus score criteria (Table 7). Visual outcome in BCVA was very similar to Ikervis^®^ therapy (Table 12).

### 3.7. Comparison of Clinical Parameters of Patients with Tolerance vs. Patients with Intolerance of Topical Ciclosporine A as 0.1% Cationic Emulsion

Considering the group of patients who tolerated Ikervis ^®^, we saw stable BCVA (mean ΔBCVA logMAR = −0.002, SD logMAR = 0.14) with 56% having an improved or stable BCVA. In the group of non-tolerating Ikervis^®^ patients, we had similar results (mean ΔBCVA logMAR = −0.02; SD logMAR = 0.089), with 67% having an improved or stable BCVA. However, “fixed deficits” such as corneal scarring or thinning have to be kept in mind as they have an impact on BCVA. In 65.33% of the patients treated with Ikervis^®^ (49/75), we found a better or at least stable BCVA, while 34.67% of patients (26/75) suffered from loss of BCVA during treatment. A similar ratio was found for AF treatment, with 65% of patients (13/20) reporting better or stable BCVA vs. 35% of patients (7/20) with visual deterioration (Table 12).

## 4. Discussion

Almost seventy percent of patients with ocular cGVHD in our cohort were on systemic immunosuppressive or immunomodulatory therapy prescribed by the treating hemato-oncologists, mainly because of GVHD of other organ systems such as the oral mucosa and skin. However, most patients had moderate to severe ocular cGVHD with ongoing or recurrent inflammatory activity despite systemic immunosuppressive treatment. Ongoing inflammation on the ocular surface indicates that ocular cGVHD is not well controlled, increases the risk of complications, and may require an intensified anti-inflammatory treatment.

Systemic immunosuppressive and immunomodulatory therapy has an effect on ocular GVHD [17], but is not sufficient in severe cases [5]. It has been shown that the effect of systemic therapy on GVHD in specific organs such as the mouth, eyes, and genital tract is relatively low [12,18]. Therefore, topical anti-inflammatory therapy is often indicated to ameliorate symptoms and to improve ocular surface inflammation in ocular cGVHD [26].

More than ninety percent of patients in our cohort received topical cyclosporine A as part of multimodal therapy. Eighty percent needed topical steroids at least once during the follow-up period. Treatment with topical corticosteroids must be managed by an ophthalmologist to monitor putative side effects and/or ocular comorbidities such as ocular hypertension and glaucoma, cataract formation, defective epithelial healing, corneal thinning and infectious keratitis, e.g., herpes simplex. Patients with chronic GVHD are at risk for all these complications even without topical steroids due to GVHD-related immunodeficiency and prior (conditioning) treatments with chemotherapies and radiation [5].

Rimexolone and fluorometholone as alternative drugs seem to provide a lower risk of steroid-induced secondary glaucoma in comparison to prednisolone acetate. However, their therapeutic potential has not been evaluated in ocular GVHD up to now and they are not available as non-preserved drugs in Europe [5]. Non-steroidal DMARDs (disease-modifying antirheumatic drugs) such as nepafenac (Nevanac^®^) should not be given in ocular GVHD patients whenever possible due to the increased risk of corneal melting [38].

Topical cyclosporine A (CSA) as an essential tool of topical therapy for severe inflammatory ocular surface disease [26] was tolerated only in two thirds (66,67%) of patients with ocular chronic GVHD in our cohort, but seemed to be effective in reducing disease severity if tolerated. Gehlsen and colleagues demonstrated even lower rates of tolerance of topical CSA used as a commercially available 0,1% cationic emulsion in a smaller group of patients with ocular cGVHD [34]. Real-world data demonstrated that cyclosporine A 0.1% as Ikervis^®^ is effective in the management of ocular surface inflammatory diseases such as allergic eye disease, dry eye disease (DED) and other diagnoses such as mucous membrane pemphigoid/Stevens–Johnson syndrome, but drug tolerance depended on the diagnosis and also on the age of the patients [39]. In this cohort—with no ocular GVHD patients included—the intolerance rate was 16.4% [39]. The tolerance and adherence are lower in ocular GVHD patients than what has been shown in clinical studies for other ocular surface diseases, which might be related to the relatively high inflammatory activity and severe dryness in ocular chronic GVHD [5]. The frequency of intolerable ocular side effects and the reduced number of commercially available drugs—at least in Europe—are the main challenges in topical CSA treatment for ocular GVHD [34]. In order to enhance drug adherence, our strategy is to reduce the inflammatory activity initially with topical steroids and inform patients beforehand about the possible side effects of CSA such as burning, stinging, swelling, and redness and about the delayed therapeutic effect.

Topical tacrolimus—as an alternative calcineurin inhibitor—has been shown to be well tolerated and effective in ocular GVHD [40,41,42,43]. However, evidence is low due to small patient numbers and its availability is limited to a pharmacy-based formula. Up to now, other topical therapies that target specific pathogenetic molecules involved in ocular GVHD such as TGFß or IL-1 have been evaluated in clinical trials, but are not available on the market yet [12,13].

In our cohort, most of the patients had inflammatory activity of higher grades (c and d according to the GAS consensus score) and higher grades of NIH eye scores (2 and 3) before initiation of a multimodal therapy including topical calcineurin inhibitors such as cyclosporine A and/or topical steroids. A comparable high incidence of inflammatory activity in ocular cGVHD has been shown by our group before in a different cohort [32]. At the end of follow-up, most of the patients had lower grades of inflammatory activity and also lower grades of the NIH eye score than before initiation of the multimodal therapy.

In our retrospective study, most of the patients retained good visual acuity despite the high prevalence of severe ocular cGVHD. The main limitation of this study is its retrospective character and the lack of patient quality-of-life questionnaires; future prospective investigations are needed.

## 5. Conclusions

In summary, most of the patients in our cohort in a specialized tertiary care university hospital setting needed topical immunomodulatory or immunosuppressive therapy, while 68% of them were under ongoing systemic immunosuppressive therapy. Topical cyclosporine A was tolerated only by 67% of patients with ocular chronic GVHD.

Therapy for ocular chronic GVHD remains challenging. The main aims of therapy are the improvement of quality of life, prevention of ocular complications, and preservation of visual function. Future strategies may focus on the prevention of the disease, but as long as there is no effective preventive strategy, topical therapies given by experienced ophthalmologists in interdisciplinary teams are needed.

## Figures and Tables

**Table 1:** Clinical scoring of ocular chronic GvHD according to the German–Austrian–Swiss consensus conference on chronic GVHD according to [5].

**Involvement of different ocular tissues:**
a. Extent of lacrimal gland dysfunction (including Schirmer test results)
b. Involvement of the lids (e.g., blepharitis, meibomian gland dysfunction, and erythema)
c. Involvement of the conjunctiva (e.g., cicatricial conjunctivitis)
d. Involvement of the cornea (e.g., keratitis and epithelial defects)
e. Others (e.g., scleritis)
**Inflammatory activity (e.g., redness of the lid margins, hyperemia of the conjunctiva)**
a. No inflammation
b. Mild inflammation
c. Moderate inflammation:
d. Severe inflammation
**Presence of sight-threatening complications/functional impairment:**
a. Complications, e.g., corneal perforation
b. Functional impairment, e.g., reduced visual acuity
c. Secondary glaucoma

**Table 2:** Clinical scoring of ocular chronic GvHD according to the NIH eye consensus score [35].

Score 0	no symptoms
Score 1	mild symptoms of dry eye or asymptomatic signs of keratoconjunctivitis sicca
Score 2	moderate dry eye symptoms partially affecting activities of daily living, requiring drops >3 times per day or punctual plugs, without vision impairment
Score 3	severe dry eye symptoms significantly affecting activities of daily living or inability to work because of ocular symptoms or loss of vision because of KCS

**Table 3:** AlloHSCT diagnoses: Number and percentage of patients.

Reason for alloHSCT		
Leukemia 62/95		65.3%
	-AML 34/95	35.8%
	-ALL 12/95	12.6%
	-CML 9/95	9.5%
	-CLL 4/95	4.2%
	-Unspecified 3/95	3.2%
Lymphomas 16/95		16.8%
	-Multiple myeloma and plasma cell myeloma 7/95	7.4%
	-Other Non-Hodgkin lymphomas 9/95	9.5%
Myelodysplastic syndromes: 15/95		15.8%
	-Primary myelofibrosis 3/95	3.2%
	-Polycythaemia vera 1/95	1.05%
	-Paroxysmal nocturnal hemoglobinuria 1/95	1.05%
	-Unspecified 13/95	10.5%
Others (unspecified, Mastozytosis)	2/95	2.1%

**Table 4:** Manifestations of systemic cGvHD in patients with ocular cGvHD: The involved organ systems and the number of different organ systems are shown.

Number/percentage of patients with cGVHD of other organ systems	74/95	77.8%
	Skin 56/95	58.95%
	Oral or nasal mucosa 30/95	31.58%
	Intestinal tract 21/95	22.11%
	Liver 19/95	20.00%
	Broncho-pulmonal 12/95	12.63%
	Joint synovia 3/95	3.16%
	One organ system 32/95	33.68%
	Two organ systems 20/95	21.05%
	Three organ systems 11/95	11.58%
	Four organ systems 6/95	6.32%

**Table 5:** Systemic immunomodulatory and immunosuppressive therapy after alloHSCT: Different therapeutic regimes and number/percentage of patients.

Systemic immunosuppressive therapy 65/95		68.42%
Single-treatment regime 33/95		34.74%
	-Cyclosporine A 17/95	17.89%
	-Mycophenolate mofetil 2/95 -Ruxolitinib 2/95	2.11% 2.11
	-Prednisolone 9/95	9.47%
	-Sirolimus 3/95	3.16%
	-Tacrolimus 1/95	1.05%
	-Lenalidomide 1/95	1.05%
Dual therapy 27/95		30.5%
	-Ciclosporin A, Prednisolon 19/95	20.00%
	-Ciclosporin A, Mycophenolate mofetil 2/95	2.11%
	-Others 6/95	6.32%
Triple therapy 5/95		5.26%
Extracorporeal photopheresis 6/95		6.32%

**Table 6:** Ocular complications and comorbidities, related to GvHD or previous/ongoing treatments after alloHSCT: Number and percentage of patients.

Ocular Pathology	Number	%
Cataract	24/95	25.26%
-corticonuclear	12/95	12.6%
-subcapsular posterior	13/95	13.7%
-coerulean	1/95	1.05%
Uveitis	1/95	1.05%
Corneal pathology	12/95	12.6%
Herpes keratitis	6/95	6.3%
Corneal graft	2/95	2.1%
Glaucoma	4/95	4.2%
Tear duct stenosis	5/95	5.3%

**Table 7:** Changes in the inflammatory activity according to the GAS Consensus Score (ranging from a, no inflammation to d, severe inflammation) during topical CSA treatment, either with Ikervis^®^ (*n* = 75) or alternative pharmacy-based formulation (AF) (*n* = 20). At the end of follow-up, more patients had lower inflammatory activity scores than at the start of the therapy.

	Ikervis^®^		AF	
Inflammatory Activity	Start of Treatment	End Of Follow-Up	Start of Treatment	End of Follow-Up
a. no	13	30	4	7
b. mild	26	35	6	9
c. moderate	26	9	6	3
d. severe	10	1	4	1
Mean	1.44	0.75	1.5	0.9
Delta		0.69		0.6

**Table 8:** Different topical therapies in patients with ocular cGVHD: Number and percentage of patients are shown.

Diagnosis of ocular cGvHD	95/95	100%
-preservative and phosphate-free lubricating eye drops	95/95	100%
-topical steroids	76/95	80%
-Ikervis^®^	88/95	92.63%
-Ikervis^®^ until at least one follow-up examination	75/95	78.95%
-alternative pharmacy-based formulation for CSA (AF) only	4/95	4.21%
-alternative pharmacy-based formulation for CSA (AF) at least once	24/95	25.26%
-autologous serum eye drops (ASEDs)	24/95	25.26%
-ASEDs until at least one follow-up examination	22/95	23.16%
-Ikervis^®^ and ASEDs at the same time	20/24	83.33%
-punctum plugs	25/95	26.32%

**Table 9:** Duration of documented different topical therapies, shown in months with standard deviation, SD, during treatment at the Department of Ophthalmology of Charité—University Medicine Berlin.

Duration of Therapy:	Mean (Months)	SD (Months)
-overall treatment in the department	31.2804878	30.76517448
-Ikervis^®^	18.9864865	16.69198078
-Alternative pharmacy-based formulation (AF)	17	16.4468842
-ASEDs (autologous serum eye drops)	15.9130435	13.93
-ASEDs at the same time as Ikervis or AF	15.8	13.04837155
-ASEDs only without Ikervis or AF	25	19
-all topical steroids	2.46037729	
-Dexa sine SE/Dexa EDO^®^	8.425	12.20914104
-DexaPos COMOD^®^	1.14516129	1.379244466
-Dexa-Gel^®^	1.07894737	1.107844729
-Lotemax^®^	0.875	0.125
-Softacort EDO^®^	0.77777778	0.07856742
-PredniPos^®^	1	0

**Table 10:** Topical steroid use with different drugs for management of inflammatory episodes: In total, 118 treatment episodes were documented during the follow-up.

Application of:			Min.–Max. Duration of Therapy (Months)
-Dexa sine SE^®^ or Dexa EDO^®^	24/118	20.34%	0.75–43
-DexaPos COMOD^®^	34/118	28.81%	0.5–3
-Dexa-Gel^®^	44/118	37.29%	0.5–7
-Lotemax^®^	4/118	3.39%	0.75–1
-Softacort^®^	10/118	8.47%	0.75–1
-PredniPos^®^	1/118	0.85%	1
-Dexamytrex^®^ ointment	1/118	0.85%	n.a.
Topical steroid therapy for >6 weeks	14/118	11.86%	

n.a.: not apllicable.

**Table 11:** Changes in the NIH eye score (score 0 to 3) during topical CSA treatment, either with Ikervis^®^ (*n* = 75) or alternative pharmacy-based CSA formulation (AF; *n* = 16). At the end of follow-up, more patients had lower NIH eye scores than at the start of the therapy. Number of patients per score category is shown.

	Ikervis^®^		AF	
NIH Eye Score	Start of Treatment	End of Follow-Up	Start of Treatment	End of Follow-Up
0	0	3	0	0
1	14	46	8	13
2	39	22	5	3
3	22	4	3	0

**Table 12:** Changes in best corrected visual acuity (BCVA) shown as logMAR during topical CSA treatment (Ikervis^®^: *n* = 75; alternative pharmacy-based formulation, AF: *n* = 20). Number of patients with better, stable or worse BCVA is shown.

Local Therapy with	Mean BCVA at Beginning Right Eye	Mean BCVA at Beginning Left Eye	Mean BCVA at End of Treatment Right Eye	Mean BCVA at End of Treatment Left Eye	Mean Difference in BCVA	BCVA Better	BCVA Stable	BCVA Worse
Ikervis^®^	0.28948604	0.29579195	0.25245557	0.24106735	−0.0363691	31	18	26
AF	0.20670974	0.25090064	0.21163282	0.23241626	−0.0258471	8	5	7

## Data Availability

Data are available from the corresponding author upon request.
